# Supplementary material for: Nociception in Chicken Embryos, Part III: Analysis of Movements before and after Application of a Noxious Stimulus
Source: Animals (Basel). 2023 Sep 8;13(18):2859. doi: 10.3390/ani13182859 (PMC10525827; doi:10.3390/ani13182859)
Supplement: Supplementary file 1 [file animals-13-02859-s001.zip › animals-2542762-supplementary information.pdf]

Supplementary Information:

Nociception in chicken embryos Part III: Analysis of movements before and after application of a noxious stimulus

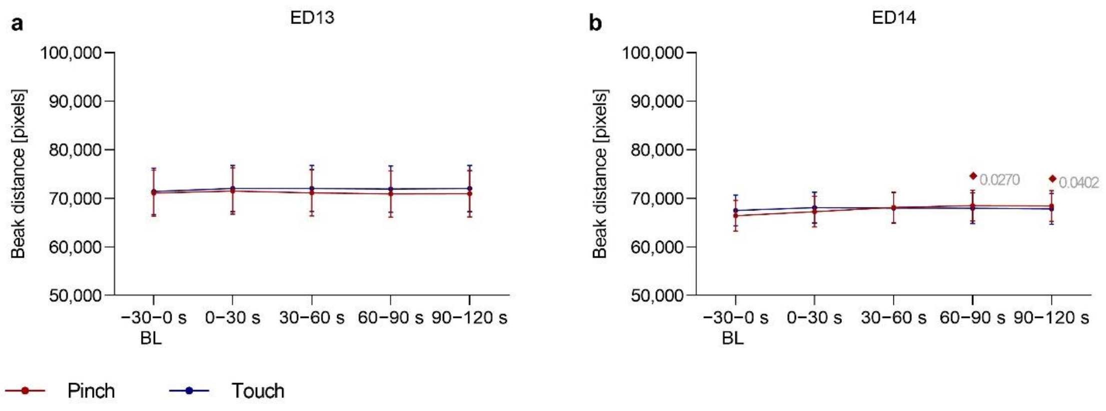

Figure S1. Beak Distance ED13/ED14.

This variable was defined as the distance between the upper and lower beak of embryos. It was measured at **a** ED13 (*n*=10) and **b** ED14 (*n* =15) before and after application of two stimuli (Touch and Pinch). The total distance in pixels across 30-second intervals (1500 frames) was evaluated. Plots show the estimated mean ± 95 % confidence intervals at the following 30-second intervals from Baseline (BL) to Post stimulation, with stimulation occurring at 0 s: -30–0, 0–30, 30–60, 60–90, and 90–120 seconds. Robust linear mixed effects were applied for all analysis. All contrasts (differences) between particular groups were assessed after model-fitting by the estimated marginal means with Tukey's P value correction for multiple comparisons. *Touch*: blue; *Pinch*: red. * Significant difference between *Pinch* and *Touch*; ♦ Significant difference from Baseline. P values shown.

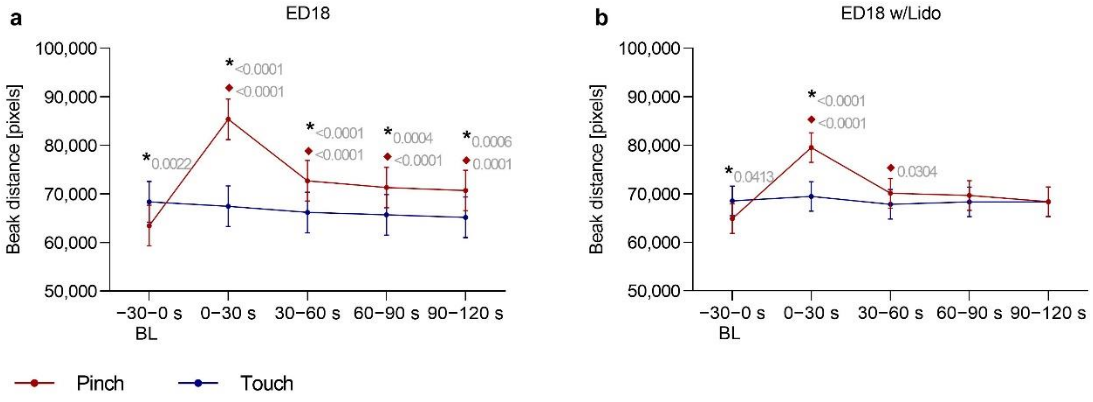

Figure S2. Beak Distance Lidocaine.

This variable was defined as the distance between the upper and lower beak of embryos. It was measured at **a** ED18 (*n*=15) and **b** ED18 w/Lido (*n*=5) before and after application of two stimuli (Touch and Pinch). The total distance in pixels across 30-second intervals (1500 frames) was evaluated. Plots show the estimated mean ± 95 % confidence intervals at the following 30-second intervals from Baseline (BL) to Post stimulation, with stimulation occurring at 0 s: -30–0, 0–30, 30–60, 60–90, and 90–120 seconds. Robust linear mixed effects were applied for all analysis. All contrasts (differences) between particular groups were assessed after model-fitting by the estimated marginal means with Tukey's P value correction for multiple comparisons. *Touch*: blue; *Pinch*: red. * Significant difference between *Pinch* and *Touch*; ♦ Significant difference from baseline. P values shown.

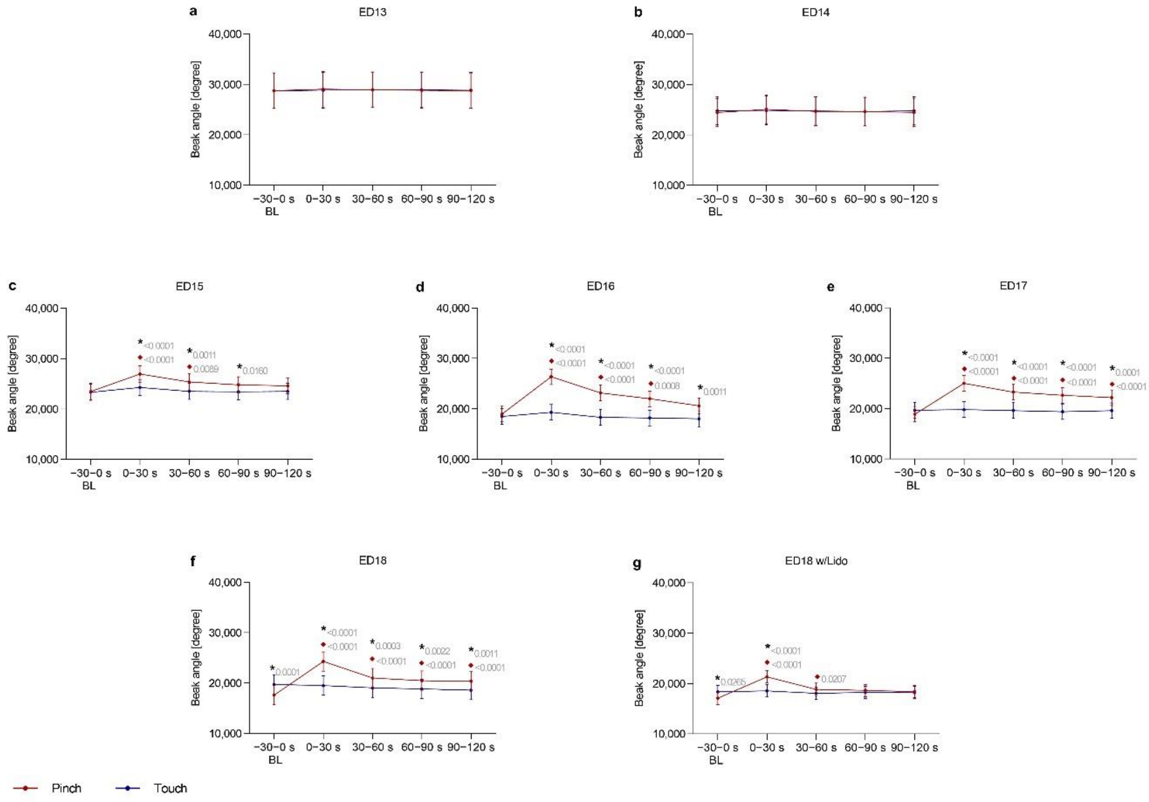

Figure S3. Beak Angle.

This variable was defined as the angle between the beak corner, the upper and lower beak of embryos. It was measured at **a** ED13 (*n*=10), **b** ED14 (*n*=15), **c** ED15 (*n*=16), **d** ED16 (*n*=16,) **e** ED17 (*n*=16), **f** ED18 (*n*=16) and **g** ED18 w/Lido (*n*=5) before and after application of two stimuli (Touch and Pinch). The total distance in pixels across 30-second intervals (1500 frames) was evaluated. Plots show the estimated mean ± 95 % confidence intervals at the following 30-second intervals from Baseline (BL) to Post stimulation, with stimulation occurring at 0 s: -30–0, 0–30, 30–60, 60–90, and 90–120 seconds. Robust linear mixed effects were applied for all analysis. All contrasts (differences) between particular groups were assessed after model-fitting by the estimated marginal means with Tukey's P value correction for multiple comparisons. *Touch*: blue; *Pinch*: red. * Significant difference between *Pinch* and *Touch*; ♦ Significant difference from baseline. P values shown.

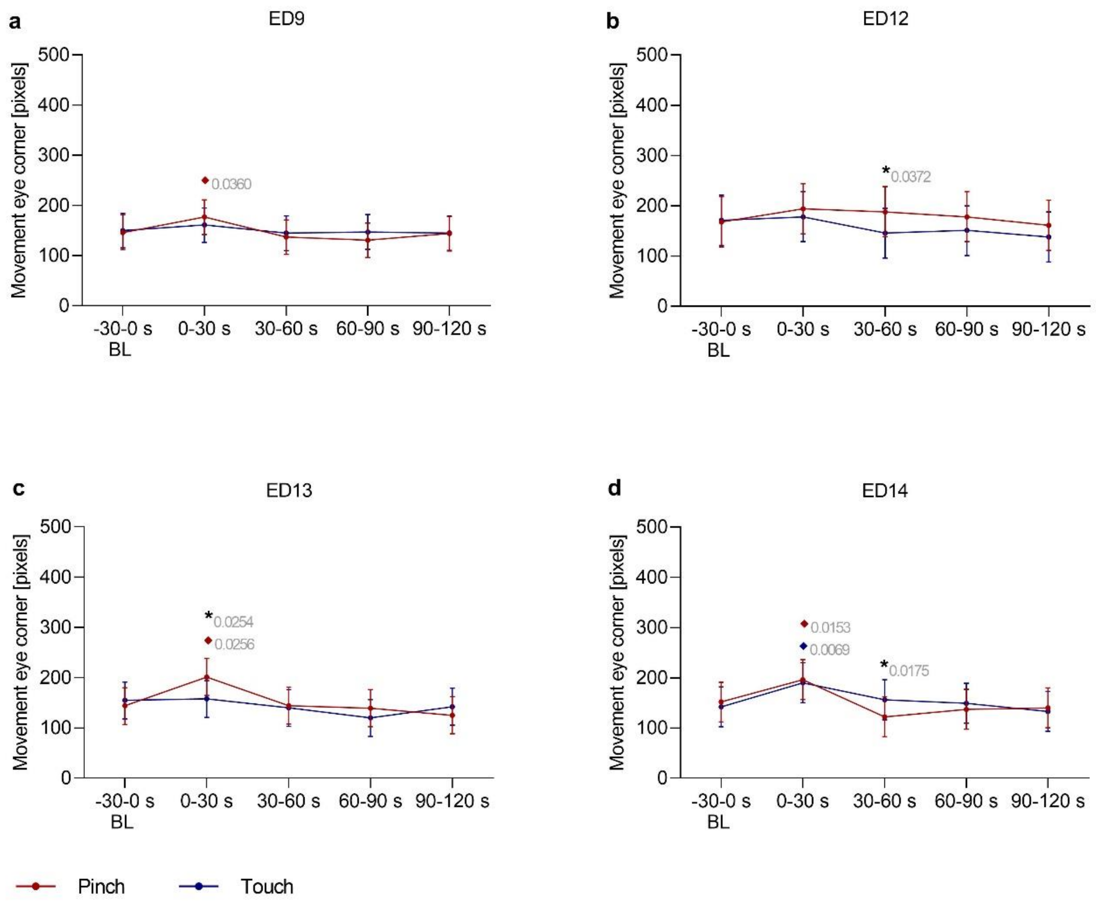

Figure S4. Movement Eye corner ED9/ED12-14.

This variable was used to detect head movements of embryos at **a** ED9 (*n*=10), **b** ED12 (*n*=10), **c** ED13 (*n*=10) and **d** ED14 (*n*=15) before and after application of two stimuli (Touch and Pinch). The total distance in pixels across 30-second intervals (1500 frames) was evaluated. Plots show the estimated mean ± 95 % confidence intervals at the following 30-second intervals from Baseline (BL) to Post stimulation, with stimulation occurring at 0 s: -30–0, 0–30, 30–60, 60–90, and 90–120 seconds. Robust linear mixed effects were applied for all analysis. All contrasts (differences) between particular groups were assessed after model-fitting by the estimated marginal means with Tukey's P value correction for multiple comparisons. *Touch*: blue; *Pinch*: red. * Significant difference between *Pinch* and *Touch*; ♦ Significant difference from baseline. P values shown.

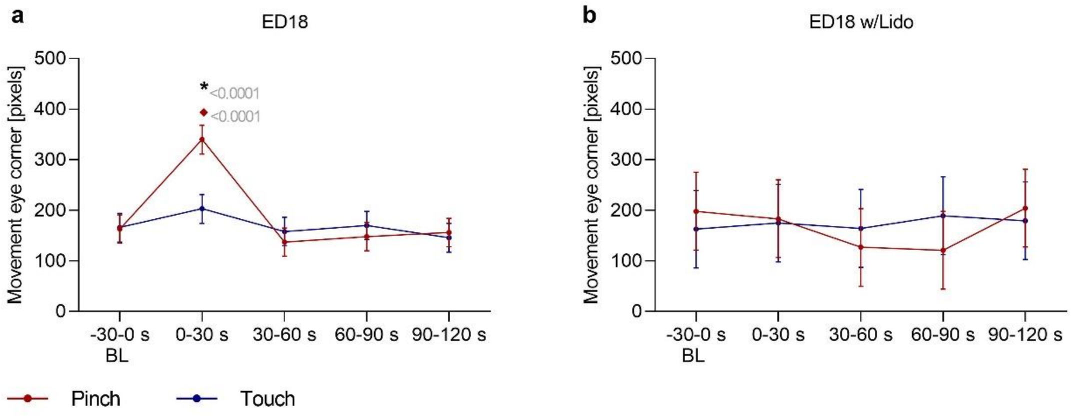

Figure S5. Movement Eye corner Lidocaine.

This variable was used to detect head movements of embryos at **a** ED18 (*n*=15) and **b** ED18 w/Lido (*n*=5) before and after application of two stimuli (Touch and Pinch). The total distance in pixels across 30-second intervals (1500 frames) was evaluated. Plots show the estimated mean ± 95 % confidence intervals at the following 30-second intervals from Baseline (BL) to Post stimulation, with stimulation occurring at 0 s: -30–0, 0–30, 30–60, 60–90, and 90–120 seconds. Robust linear mixed effects were applied for all analysis. All contrasts (differences) between particular groups were assessed after model-fitting by the estimated marginal means with Tukey's P value correction for multiple comparisons. *Touch*: blue; *Pinch*: red. * Significant difference between *Pinch* and *Touch*; ♦ Significant difference from baseline. P values shown.

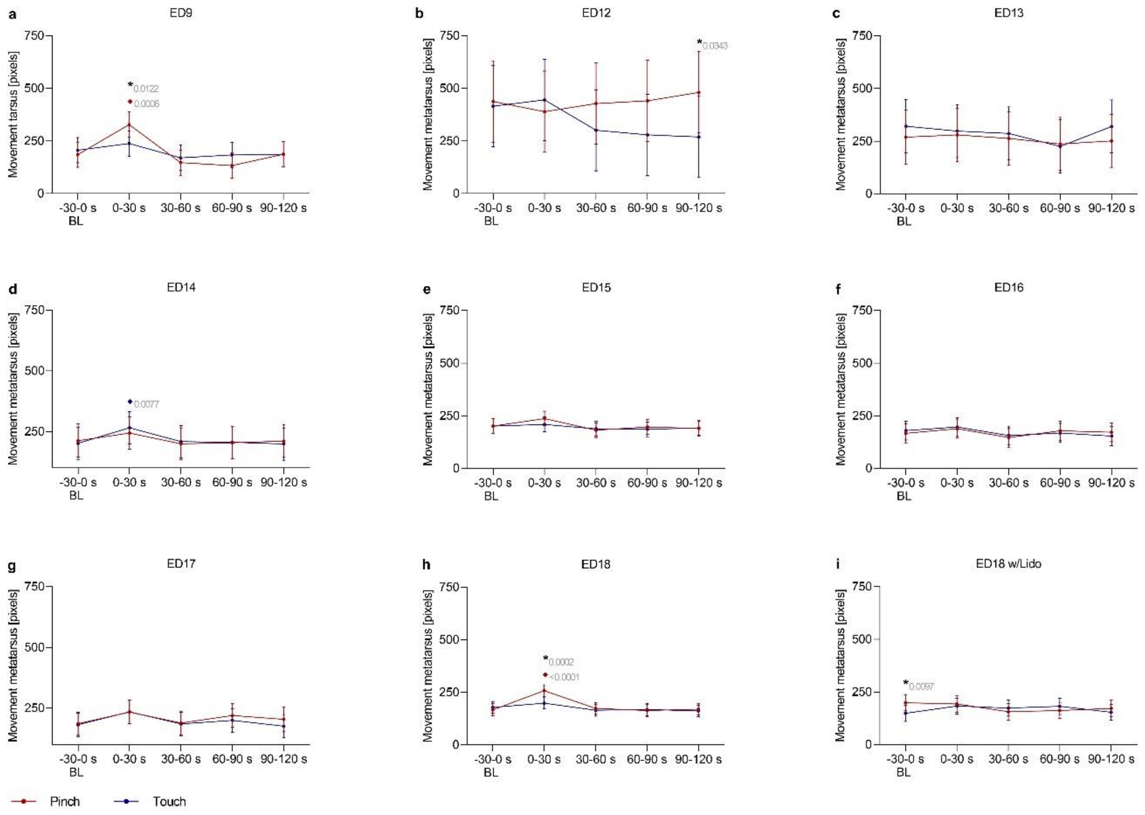

Figure S6. Movement Metatarsus respectively Tarsus (ED9).

This variable was used to detect leg movements of embryos at **a** ED9 (*n*=10), **b** ED12 (*n*=10), **c** ED13 (*n*=10), **d** ED14 (*n*=15,) **e** ED15 (*n*=16), **f** ED16 (*n*=16), **g** ED17 (*n*=16), **h** ED18 (*n*=16) and **i** ED18 w/Lido (*n*=5) before and after application of two stimuli (Touch and Pinch). The total distance in pixels across 30-second intervals (1500 frames) was evaluated. Plots show the estimated mean ± 95 % confidence intervals at the following 30-second intervals from Baseline (BL) to Post stimulation, with stimulation occurring at 0 s: -30–0, 0–30, 30–60, 60–90, and 90–120 seconds. Robust linear mixed effects were applied for all analysis. All contrasts (differences) between particular groups were assessed after model-fitting by the estimated marginal means with Tukey's P value correction for multiple comparisons. *Touch*: blue; *Pinch*: red. * Significant difference between *Pinch* and *Touch*; ♦ Significant difference from baseline. P values shown.

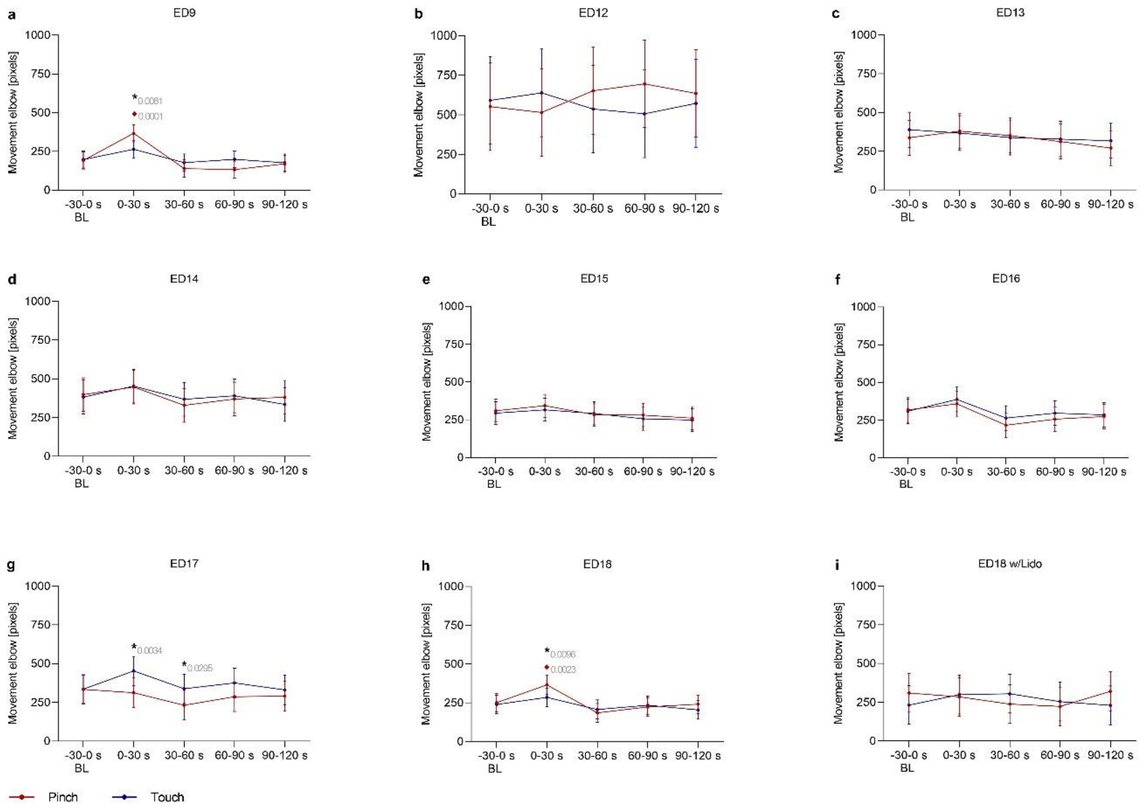

Figure S7. Movement Elbow.

This variable was used to detect wing movements of embryos at **a** ED9 (*n*=10), **b** ED12 (*n*=10), **c** ED13 (*n*=10), **d** ED14 (*n*=15,) **e** ED15 (*n*=16), **f** ED16 (*n*=16), **g** ED17 (*n*=16), **h** ED18 (*n*=16) and **i** ED18 w/Lido (*n*=5) before and after application of two stimuli (Touch and Pinch). The total distance in pixels across 30-second intervals (1500 frames) was evaluated. Plots show the estimated mean ± 95 % confidence intervals at the following 30-second intervals from Baseline (BL) to Post stimulation, with stimulation occurring at 0 s: -30–0, 0–30, 30–60, 60–90, and 90–120 seconds. Robust linear mixed effects were applied for all analysis. All contrasts (differences) between particular groups were assessed after model-fitting by the estimated marginal means with Tukey's P value correction for multiple comparisons. *Touch*: blue; *Pinch*: red. * Significant difference between *Pinch* and *Touch*; ♦ Significant difference from baseline. P values shown.

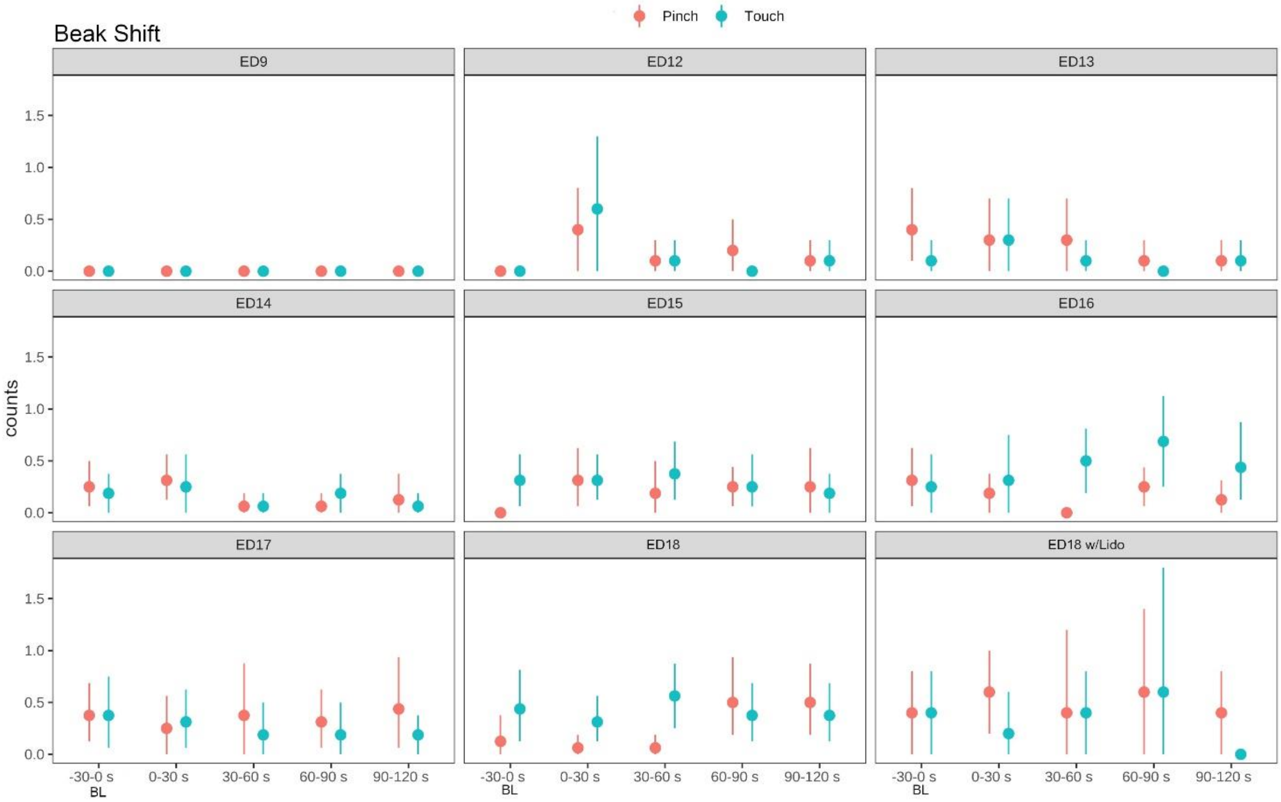

Figure S8. Beak Shift.

The variable was used to detect the number of times (counts) embryos at ED9 (*n*=10), ED12 to ED13 (*n*=10), ED14 to ED18 (*n*=16) and ED18 w/ Lido (*n*=5) showed the behavior *Beak Shift* before and after stimulus (*Touch*/*Pinch*). A nonparametric bootstrap for obtaining confidence limits for the mean without assuming normality was used for the visualization of data.

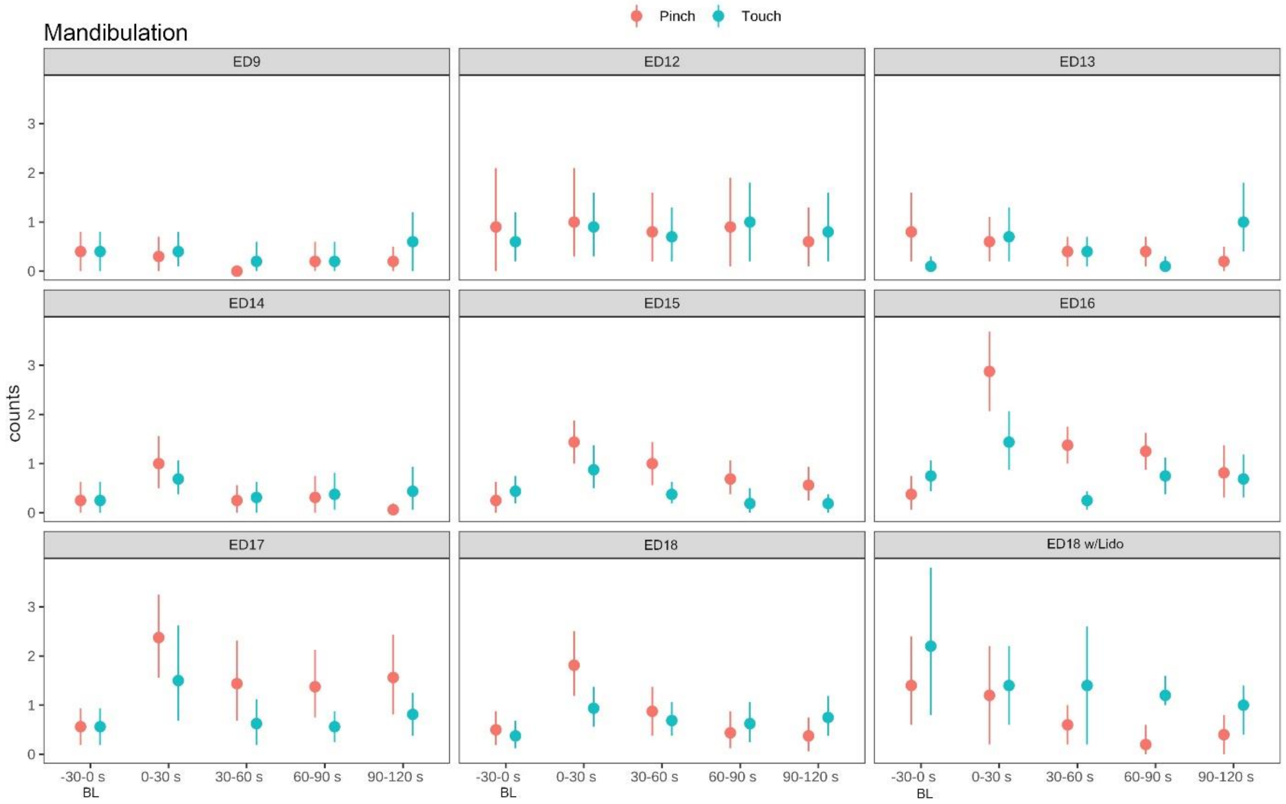

Figure S9. Mandibulation.

The variable was used to detect the number of times (counts) embryos at ED9 (*n*=10), ED12 to ED13 (*n*=10), ED14 to ED18 (*n*=16) and ED18 w/ Lido (*n*=5) showed the behavior *Mandibulation* before and after stimulus (*Touch*/*Pinch*). A nonparametric bootstrap for obtaining confidence limits for the mean without assuming normality was used for the visualization of data.

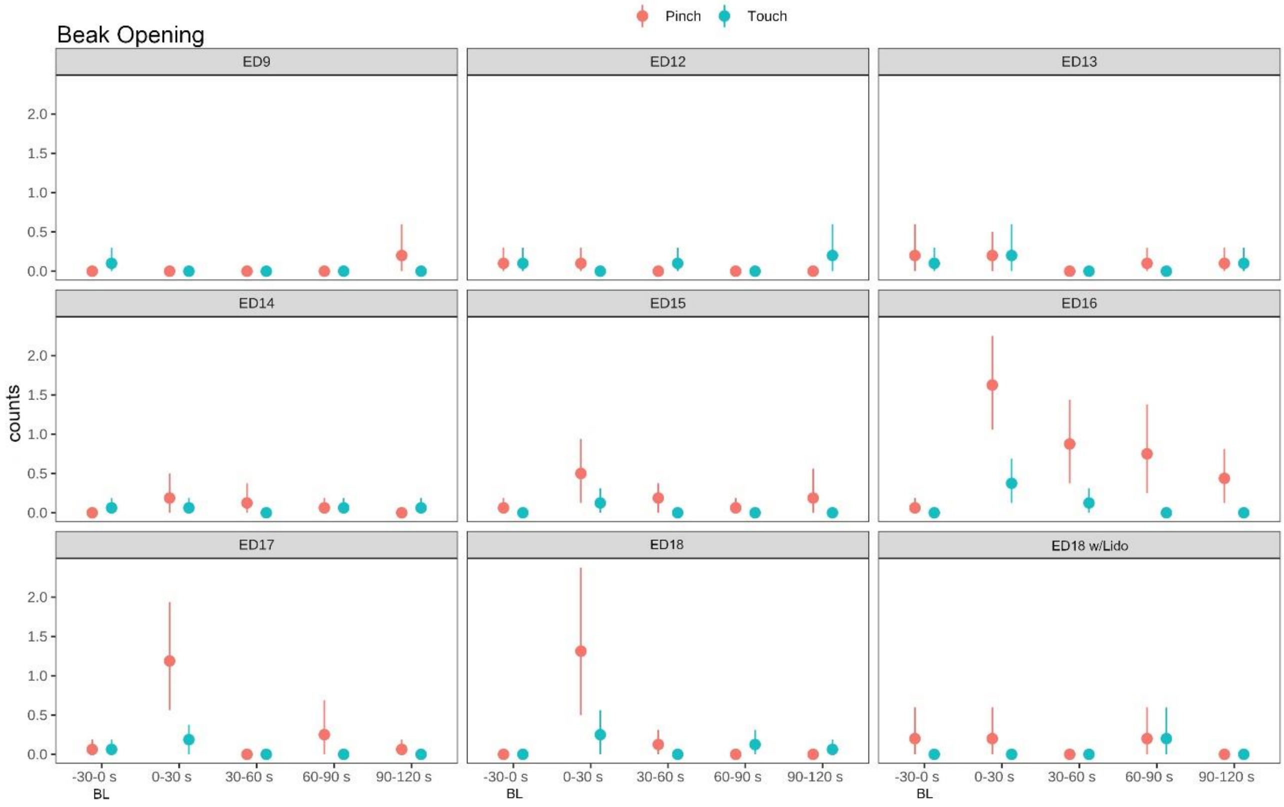

Figure S10. Beak Opening.

The variable was used to detect the number of times (counts) embryos at ED9 (n=10), ED12 to ED13 (n=10), ED14 to ED18 (n=16) and ED18 w/ Lido (n=5) showed the behavior *Beak Opening* before and after stimulus (*Touch*/*Pinch*). A nonparametric bootstrap for obtaining confidence limits for the mean without assuming normality was used for the visualization of data.

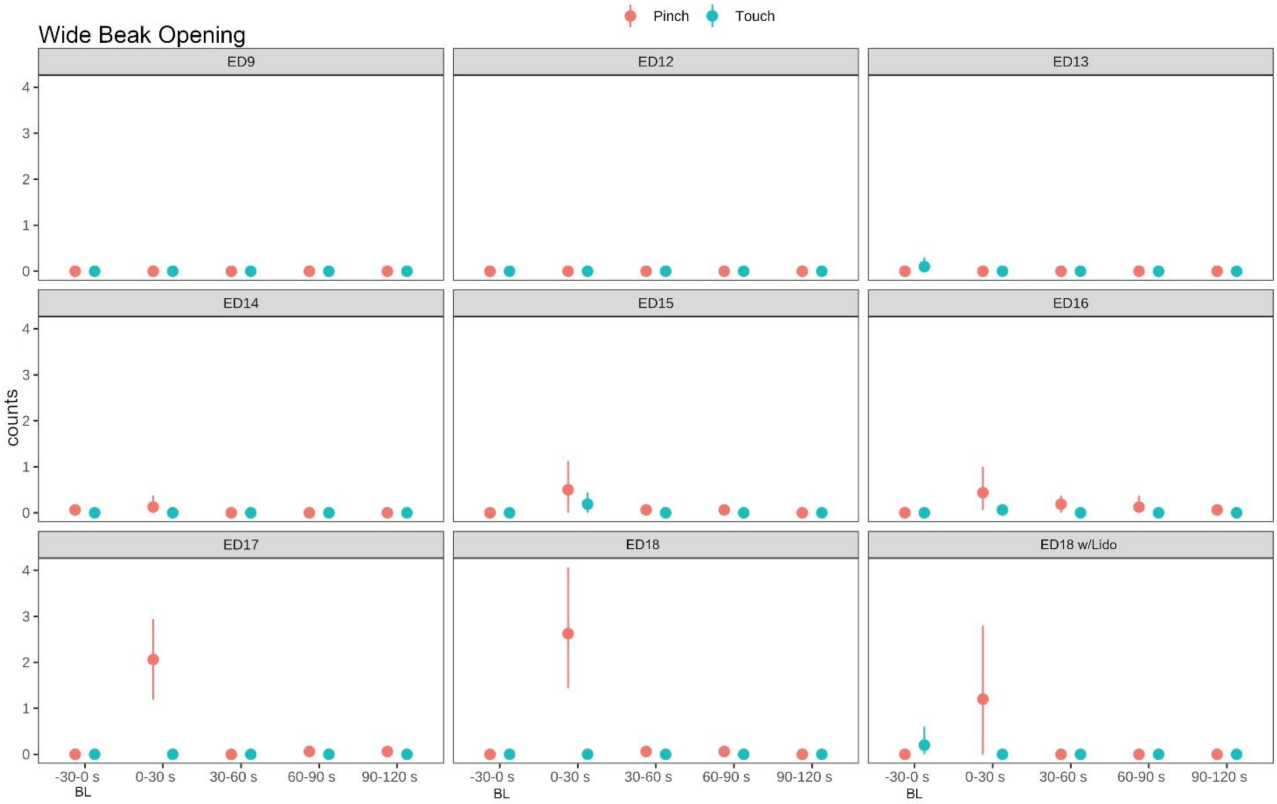

Figure S11. ***Wide Beak Opening.***

The variable was used to detect the number of times (counts) embryos at ED9 (n=10), ED12 to ED13 (n=10), ED14 to ED18 (n=16) and ED18 w/ Lido (n=5) showed the behavior *Wide Beak Opening* before and after stimulus (*Touch*/*Pinch*). A nonparametric bootstrap for obtaining confidence limits for the mean without assuming normality was used for the visualization of data.

Table S1. Overview on the number of datasets from DLC included in the final analysis.

**ED18**						
	**Time Interval**	**Beak angle**	**Beak distance**	**Elbow**	**Eye corner**	**Metatarsus**
**Touch**	−30–0 sec	15	15	15	15	14
	0–30 sec	15	15	15	15	14
	30–60 sec	15	15	15	15	14
	60–90 sec	15	15	15	15	14
	90–120 sec	15	15	15	15	14
**Pinch**	−30–0 sec	15	15	15	15	14
	0–30 sec	15	15	15	15	14
	30–60 sec	15	15	15	15	14
	60–90 sec	15	15	15	15	14
	90–120 sec	15	15	15	15	14
**ED17**						
	**Time Interval**	**Beak angle**	**Beak distance**	**Elbow**	**Eye corner**	**Metatarsus**
**Touch**	−30–0 sec	16	16	16	16	16
	0–30 sec	15	16	16	16	16
	30–60 sec	16	16	15	16	16
	60–90 sec	15	16	15	16	16
	90–120 sec	15	16	15	16	16
**Pinch**	−30–0 sec	16	16	16	16	16
	0–30 sec	15	16	15	16	15
	30–60 sec	15	16	15	16	15
	60–90 sec	16	15	15	16	15
	90–120 sec	16	16	15	16	16
**ED16**						
	**Time Interval**	**Beak angle**	**Beak distance**	**Elbow**	**Eye corner**	**Metatarsus**
**Touch**	−30–0 sec	16	16	16	16	16
	0–30 sec	16	16	16	16	16
	30–60 sec	16	16	16	16	16
	60–90 sec	16	16	16	16	16
	90–120 sec	15	16	16	16	16
**Pinch**	−30–0 sec	16	16	16	16	16
	0–30 sec	16	16	16	16	16
	30–60 sec	16	16	16	16	16
	60–90 sec	16	16	16	16	16
	90–120 sec	16	16	16	16	16

ED15						
	**Time Interval**	**Beak angle**	**Beak distance**	**Elbow**	**Eye corner**	**Metatarsus**
**Touch**	−30–0 sec	16	16	16	16	16
	0–30 sec	16	16	16	16	16
	30–60 sec	16	16	16	16	16
	60–90 sec	16	16	16	16	16
	90–120 sec	15	16	16	16	16
**Pinch**	−30–0 sec	16	16	16	16	16
	0–30 sec	16	16	16	16	16
	30–60 sec	16	16	16	16	16
	60–90 sec	16	16	16	16	16
	90–120 sec	16	16	16	16	16
**ED14**						
	**Time Interval**	**Beak angle**	**Beak distance**	**Elbow**	**Eye corner**	**Metatarsus**
**Touch**	−30–0 sec	15	15	15	15	13
	0–30 sec	15	15	15	15	13
	30–60 sec	15	15	15	15	13
	60–90 sec	15	15	15	15	13
	90–120 sec	15	15	15	15	13
**Pinch**	−30–0 sec	15	15	15	15	13
	0–30 sec	15	15	15	15	13
	30–60 sec	15	15	15	15	13
	60–90 sec	15	15	15	15	13
	90–120 sec	15	15	15	15	13
**ED13**						
	**Time Interval**	**Beak angle**	**Beak distance**	**Elbow**	**Eye corner**	**Metatarsus**
**Touch**	−30–0 sec	10	10	10	10	8
	0–30 sec	10	10	10	10	9
	30–60 sec	10	10	10	10	9
	60–90 sec	10	10	10	10	9
	90–120 sec	10	10	10	10	9
**Pinch**	−30–0 sec	10	10	10	10	9
	0–30 sec	10	10	10	10	9
	30–60 sec	10	10	10	10	9
	60–90 sec	10	10	10	10	9
	90–120 sec	10	10	10	10	9

ED12						
	**Time Interval**	**Beak angle**	**Beak distance**	**Elbow**	**Eye corner**	**Metatarsus**
**Touch**	−30–0 sec	10	10	9	10	8
	0–30 sec	10	10	9	10	8
	30–60 sec	10	10	9	10	8
	60–90 sec	10	10	9	10	8
	90–120 sec	10	10	9	10	8
**Pinch**	−30–0 sec	10	10	9	10	8
	0–30 sec	10	10	9	10	8
	30–60 sec	10	10	9	10	8
	60–90 sec	10	10	9	10	8
	90–120 sec	10	10	9	10	8
**ED9**						
	**Time Interval**	**Beak angle**	**Beak distance**	**Elbow**	**Eye corner**	**Tarsus**
**Touch**	−30–0 sec	10	10	10	10	10
	0–30 sec	10	10	10	10	10
	30–60 sec	10	10	10	10	10
	60–90 sec	10	10	10	10	10
	90–120 sec	10	10	10	10	10
**Pinch**	−30–0 sec	10	10	10	10	10
	0–30 sec	10	10	10	10	10
	30–60 sec	10	10	10	10	10
	60–90 sec	10	10	10	10	10
	90–120 sec	10	10	10	10	10
**ED18 w/ Lido**						
	**Time Interval**	**Beak angle**	**Beak distance**	**Elbow**	**Eye corner**	**Metatarsus**
**Touch**	−30–0 sec	5	5	5	5	5
	0–30 sec	5	5	5	5	5
	30–60 sec	5	5	5	5	5
	60–90 sec	5	5	5	5	5
	90–120 sec	5	5	5	5	5
**Pinch**	−30–0 sec	5	5	5	5	5
	0–30 sec	5	5	5	5	5
	30–60 sec	5	5	5	5	5
	60–90 sec	5	5	5	5	5
	90–120 sec	5	5	5	5	5
